# Surface-plasmon-enhanced ultraviolet emission of Au-decorated ZnO structures for gas sensing and photocatalytic devices

**DOI:** 10.3762/bjnano.9.70

**Published:** 2018-03-01

**Authors:** T Anh Thu Do, Truong Giang Ho, Thu Hoai Bui, Quang Ngan Pham, Hong Thai Giang, Thi Thu Do, Duc Van Nguyen, Dai Lam Tran

**Affiliations:** 1 Institute of Materials Science, Vietnam Academy of Science and Technology, 18 Hoang Quoc Viet, Caugiay, 100000, Hanoi, Vietnam; 2 Petro Vietnam University, 762 Cach Mang Thang 8, Longtoan, 790000, Ba Ria-Vung Tau, Vietnam; 3 Graduate University of Science and Technology, Vietnam Academy of Science and Technology, 18 Hoang Quoc Viet, Caugiay, 100000, Hanoi, Vietnam

**Keywords:** Au-decorated ZnO, carrier dynamics, gas sensors, photocatalyst, SPR effect

## Abstract

Pure and Au-decorated sub-micrometer ZnO spheres were successfully grown on glass substrates by simple chemical bath deposition and photoreduction methods. The analysis of scanning electron microscopy (SEM) and transmission electron microscopy (TEM) images, energy-dispersive X-ray spectroscopy (EDS), UV–vis absorption, and photoluminescence (PL) spectra results were used to verify the incorporation of plasmonic Au nanoparticles (NPs) on the ZnO film. Time-resolved photoluminescence (TRPL) spectra indicated that a surface plasmonic effect exists with a fast rate of charge transfer from Au nanoparticles to the sub-micrometer ZnO sphere, which suggested the strong possibility of the use of the material for the design of efficient catalytic devices. The NO_2_ sensing ability of as-deposited ZnO films was investigated with different gas concentrations at an optimized sensing temperature of 120 °C. Surface decoration of plasmonic Au nanoparticles provided an enhanced sensitivity (141 times) with improved response (τ_Res_ = 9 s) and recovery time (τ_Rec_ = 39 s). The enhanced gas sensing performance and photocatalytic degradation processes are suggested to be attributed to not only the surface plasmon resonance effect, but also due to a Schottky barrier between plasmonic Au and ZnO structures.

## Introduction

Inorganic transition metal oxide sensor devices have attracted attention in particular for improving gas sensing, energy conversion, electronics, photocatalysis and optoelectronic devices [1–4]. Among them, ZnO nanostructures have particularly attracted attention for use in gas sensors due to their stability and relatively high sensitivity to target gases such as NO_2_, NO, CO, *n*-propane (C_3_H_8_), and NH_3_. In general, gas sensing devices based on ZnO structures are evidenced to be influenced by many factors, including absorbed oxygen species (O^2−^, O^−^, and O_2_^−^), charge carrier concentration, and the defects and vacancies on the ZnO surfaces. The gas response performance can be improved by a charge transfer process through surface modification [3,5–6] and doping of catalytic materials [7–9]. The quantity of active electrons involved in the surface response plays a key role for the sensor performance. The incorporation of noble metal nanostructures such as gold (Au) into ZnO surfaces is considered as an effective way to enhance the gas sensing response and to reduce the operation temperature and response/recovery times. The various origins of these properties are commonly assigned to the following two phenomena: (i) a surface plasmon resonance (SPR) effect of plasmonic gold nanoparticles (Au NPs) could certainly take place and contribute to the electrical transport behavior for Au-decorated ZnO [10–11]. SPR of Au NPs strongly depends upon the Au–ZnO matrix interface, as well as the dielectric properties of the surrounding ZnO matrix [12–13]. (ii) The AuNPs possibly deplete more carriers near the ZnO surface, which increases the charge density of ZnO and leads to enhanced interaction with target gases [14–16].

In another research strategy to obtain high photocatalytic performance materials, ZnO, a wide-band gap semiconductor (*E*_g_ > 3 eV), can also be used as a suitable material for photocatalysts based on the particular plasmonic characteristics of the nanostructures. Herein, it was found that the synthesis of very diverse ZnO structures was easily controllable by different methods, for example, using hydrothermal routes. Thus, ZnO nano- or even microstructures decorated by some noble metals (Au, Ag and Pt) to form plasmonic metal/semiconductor structures are very attractive and are expected to serve as a potential photocatalysts with highly effective performance. For instance, plasmonic Au NP/vertically aligned ZnO nanorod structures were proposed for water splitting, solar cells, and environmental remediation [17–19]. Ag/ZnO nanohybrid structures were synthesized and investigated for photocatalytic activity as reported previously [8,20]. The photocatalytic activity of the Pt/ZnO hybrid nanocomposite under photodegradation of rhodamine B (RhB) was higher compared to commercial TiO_2_ [21]. Here, it is quite reasonable to note that the plasmonic metal NP/metal-oxide semiconductor structures are also promising and interesting materials for photocatalytic utilization, in particular relating to the solar light spectrum. It is also worth mentioning that, in fact, there are numerous works that have investigated the photocatalytic activity of these structures based on microstructured oxides and that they exhibited an even higher effect than those of nanostructured oxides. Most recently, the microstructured oxide structures with particular surface morphology have been examined for their exciting characteristics [22–23]. Thus, plasmonic structures with micrometer-sized metal-oxide semiconductors are still of significant consideration.

In this work, we propose a simple photochemical approach to synthesize Au NPs directly deposited on the surface of pre-synthesized ZnO nanostructures synthesized by chemical bath deposition on glass substrates. Morphological evaluation revealed that ZnO sub-micrometer spheres were deposited on the glass substrates and Au-decorated ZnO nanostructures were confirmed. Using photoluminescence (PL) spectra and time-resolved photoluminescence (TRPL) measurements, we investigate the SPR characteristics and ZnO exciton peaks, which complement and support the design of efficient NO_2_ gas sensing and photocatalysis devices. The effects of Au NPs on the performance of ZnO-based gas sensors were also investigated at an optimized operating temperature of 120 °C towards pollutant gases in a wide range of concentrations. The enhanced sensitivity and reasonably fast response/recovery time were reported for a gas sensor based on Au-decorated ZnO structures. The highest selectivity towards NO_2_ was compared to other combustion gases such as CO, and C_3_H_8_. In addition, the photocatalytic decomposition of organic dyes under sunlight using PL quenching measurements shows that the Au-decorated ZnO structures exhibited an enhancement in electron−hole pair separation, which allows for superior photocatalytic activity.

## Results and Discussion

**Figure 1:**
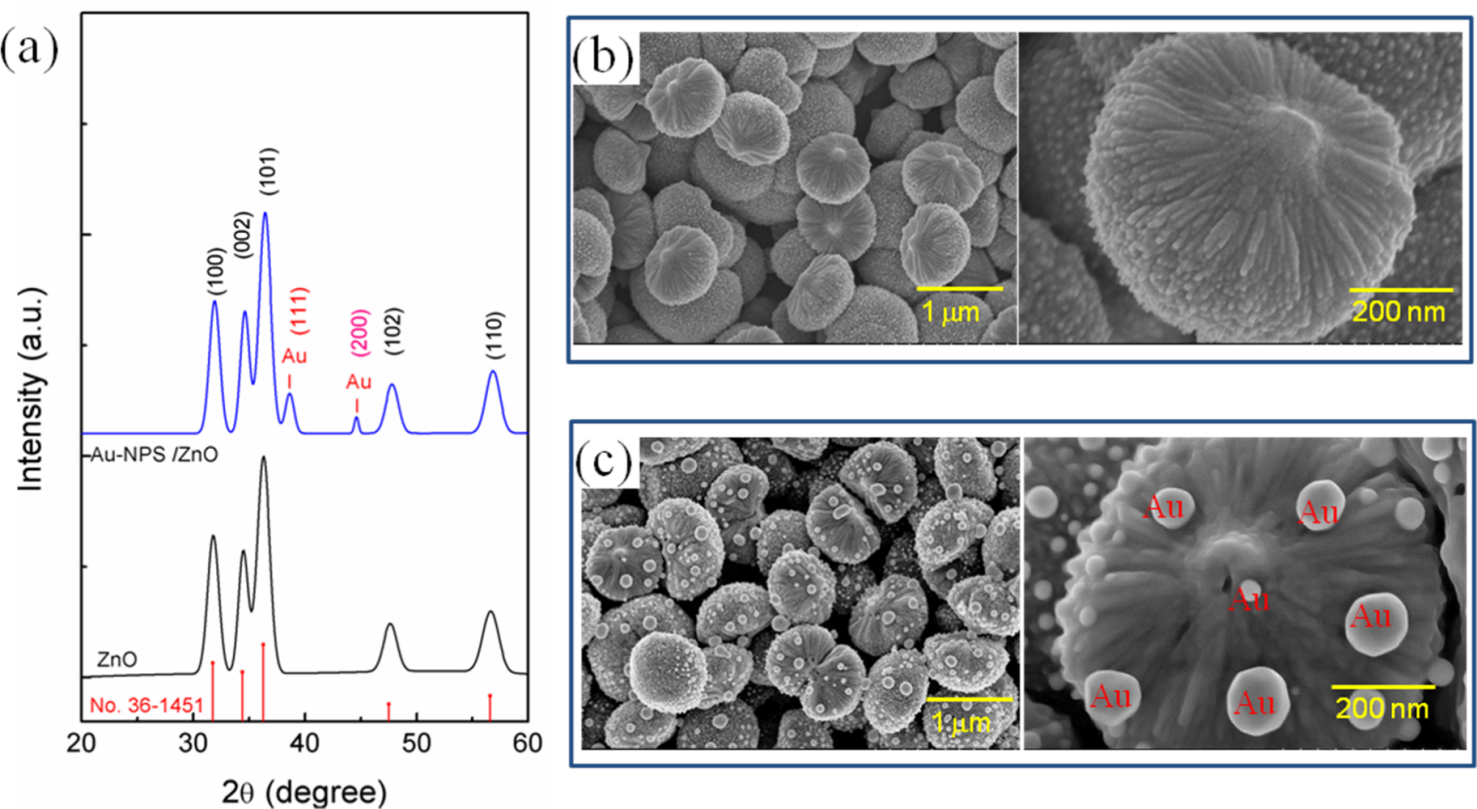
XRD patterns (a), FE-SEM images of as-deposited ZnO and Au nanoparticle/ZnO structures using the chemical bath deposition approach (b and c).

X-ray diffraction (XRD) patterns of all samples are shown in Figure 1a. For both pure and Au-decorated ZnO, a hexagonal wurtzite ZnO phase was found with peaks well indexed to the JCPDS card No. 36-1451. For the Au NP/ZnO sample, there were two additional diffraction peaks located at 2θ = 38.6 and 44.6° that can be assigned to the (111) and (200) planes of the face-centered cubic structured Au (JCPDS No. 4-784). This indicated that Au nanoparticles were deposited on the surface of ZnO sub-micrometer spheres, which can be seen more clearly in FE-SEM images. As shown in Figure 1b,c, the uniformly shaped ZnO sub-micrometer spheres with an average diameter of about 0.8 μm were dispersed on the glass substrates. The higher magnification clearly shows that each sub-micrometer sphere is assembled from numerous nanorods pointing toward the center of the microsphere.

**Figure 2:**
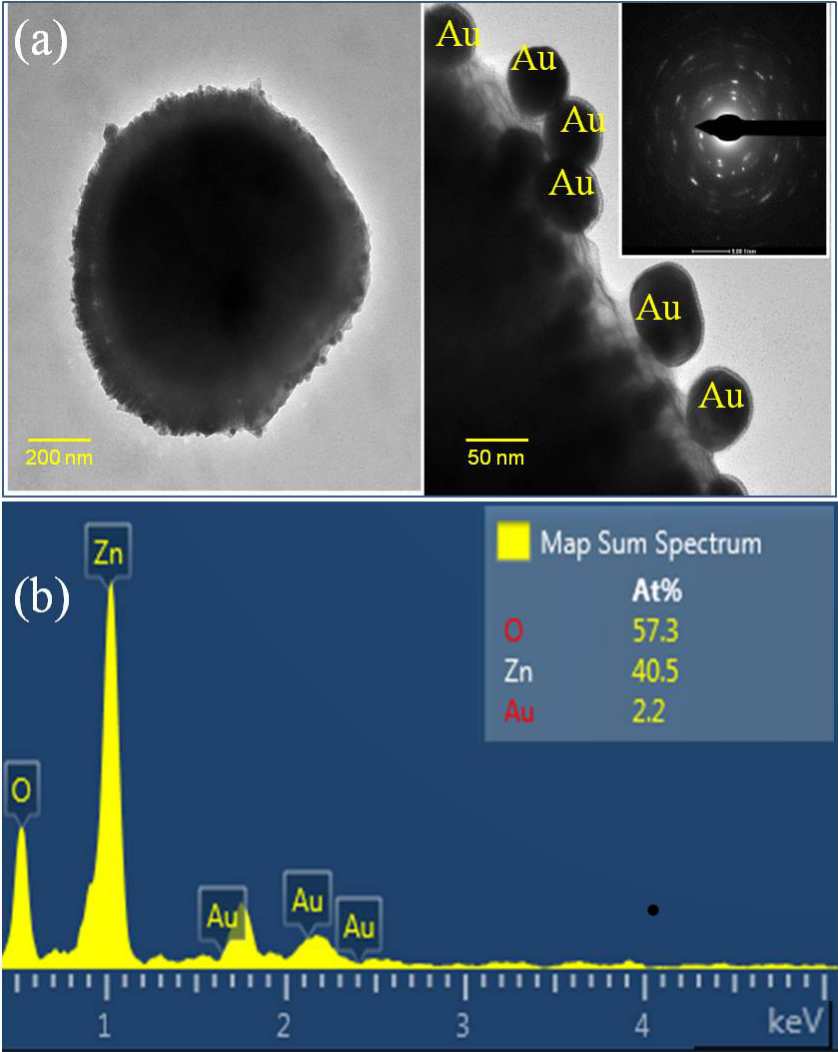
(a) TEM images and (b) EDS spectrum of Au nanoparticle/ZnO structures.

Figure 1c reveals the formation of spherically shaped Au nanopaticles on the surface of ZnO sub-micrometer spheres. Au nanoparticles were adsorbed on the surface of ZnO sub-micrometer spheres due to the photogenerated electrons from ZnO under UV illumination that reacted with AuCl_4_^−^ ions. Figure 2a shows the high-resolution TEM images of Au NPs incorporated in ZnO at different magnifications. The observed size distribution of the Au NPs is in the range of 20–50 nm. It can be assumed that Au nanoparticles were decorated on the ZnO sub-micrometer spheres due to the strong electrostatic attraction. The presence of a gold element in the ZnO matrix was confirmed by elemental analysis from energy-dispersive X-ray spectroscopy (EDS), as shown in Figure 2b. The formation of such a ZnO sub-micrometer sphere suggests that the acetate ions from the precursor salt can be helpful ZnO seeds, which grow preferentially along the c-axis during the thermal treatment. Mono-ethanolamine (MEA) molecules not only act as a dispersant to prevent particle agglomeration, but also bind to the crystal seed surface. These are believed to play an important role in the weakening of grain growth and the assembly of nanorods. Owing to the stronger bonding between the active component of the deposition solution and substrate surface, the self-assembly of the nanorod precursors is higher, resulting in the core-spike architecture by minimizing the surface energy.

It was found that the plasmonic metal nanomaterial/metal-oxide semiconductor structures were preferred as the nanometer-sized metal oxides for high photocatalytic activity and wide dispersion. However, in this manner, the hot electron under photoexcitation pumping from the plasmonic metal to the semiconductor can be limited due to the high depletion barrier of the metal-oxide surfaces [1], resulting in the reduction of photocatalytic activity. From the above results, the synthesized ZnO microsphere particles with rough surface morphology could provide an advantage for enhancing the plasmonic effect when incorporated with Au NPs, which showed great performance in gas sensing and photocatalytic decomposition of organic pollution substances [14–16,22–25].

**Figure 3:**
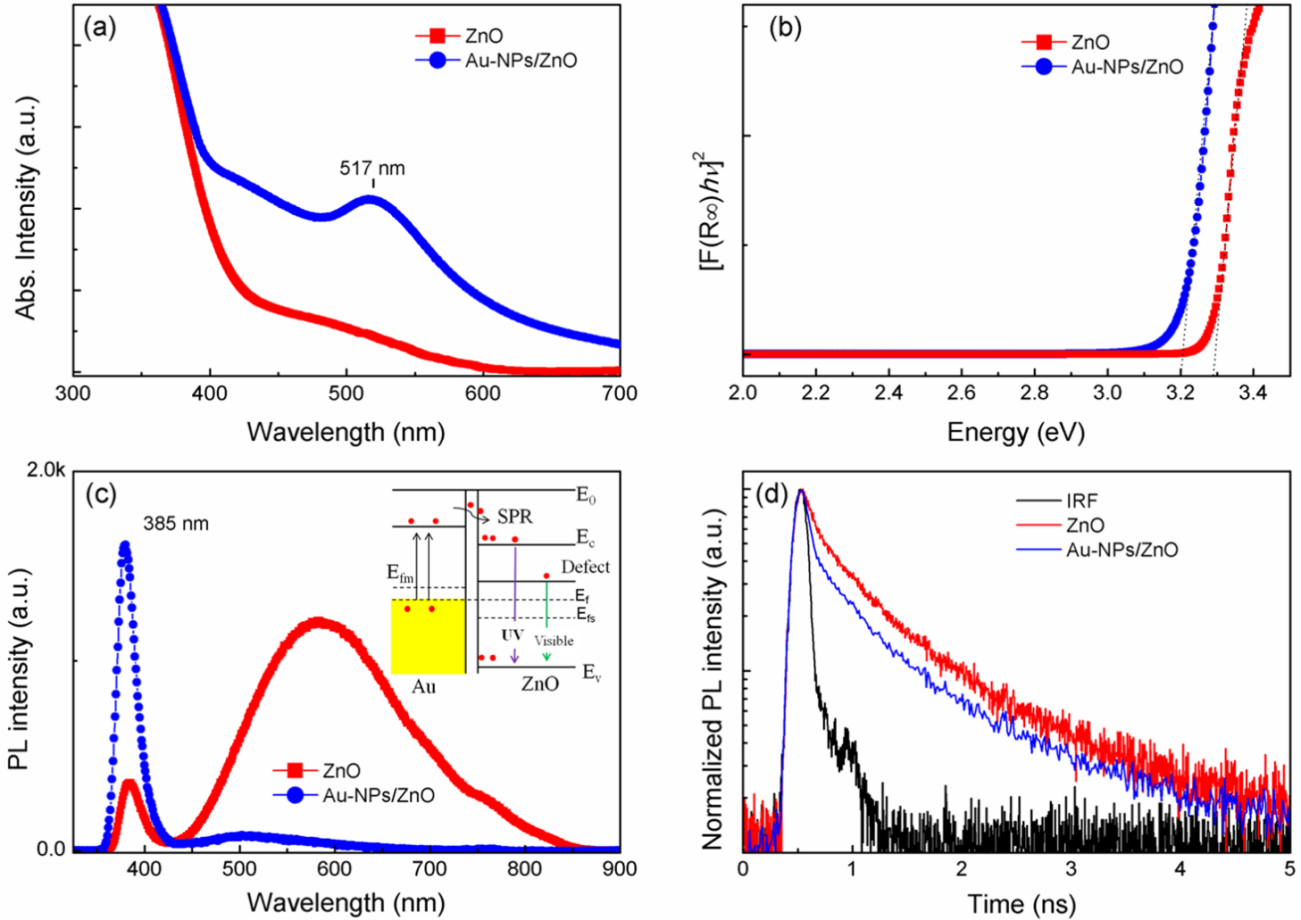
(a) Absorption spectra; (b) Kubelka–Munk transformed spectra; (c) photoluminescence spectra at room temperature; and (d) time-resolved photoluminescence spectra at room temperature of as-deposited ZnO and Au NP/ZnO films. The instrument response function (IRF) was less than 130 ps.

Figure 3a shows the UV–vis absorption spectra of ZnO and Au NP/ZnO films. A typical absorption peak in the UV region around 365 nm is observed on the pure ZnO as well as on the Au NP/ZnO sample, which originates from the band edge absorption of ZnO. However, the Au NP/ZnO sample exhibits an extra peak centered at 517 nm, corresponding to the surface plasmon resonance band of Au NPs, which further confirms the formation of the hybrid Au NP/ZnO structures [24–25]. Using the Kubelka–Munk function and Tauc plots, the band gaps (*E*_g_) were determined as 3.3 and 3.2 eV for ZnO and Au NPs/ZnO, respectively, as shown in Figure 3b. It can be seen that the band gap of the ZnO particles slightly decreases when decorated with the Au NPs.

Photoluminescence measurements of ZnO and Au NP/ZnO samples were performed at room temperature, as shown in Figure 3c. The PL spectra exhibit an intense, narrow UV band edge emission at 385 nm, and a defect-related emission band at 600 nm which was attributed to defects and vacancies [5,9,26–28]. After the Au NPs were decorated on the ZnO sub-micrometer spheres, one can see an enhanced band edge emission and a quenched visible emission. Interestingly, the UV–vis spectrum of plasmonic Au NPs overlaps with the defect-related emission band of ZnO. The electron–hole (e–h) pairs near the Au NP/ZnO interface generated by the laser source are extracted by the local electric field in the region of the energy barrier of the Au NP/ZnO structure, thus increasing the free carrier density and reducing the energy barrier. The inset of Figure 3c shows the band bending, the Fermi energy level of the ZnO and the electron transfer from Au to ZnO. The e–h recombination in Au NP/ZnO structures can be promoted and leads to an enhanced UV emission. The strong electronic interaction between Au NPs and the defect sites of ZnO can be caused by near-surface resonance coupling of ZnO, which results in the quenching of the defect-related emission band. As a result, the band edge emission is enhanced and visible emission is quenched similar to previously reported works [1,29–32]. Moreover, in order to obtain insightful information for depicting the fast charge carriers as well as to elucidate the mechanism of charge transfer, TRPL spectra were recorded at room temperature for all samples (Figure 3d). The fast decay time (fast and slow), as extracted by fitting the bi-exponential curve at the 385 nm emission peak, was equal to 150 ps and 995 ps for Au NP/ZnO samples, respectively. This is comparable to the values for ZnO found to be 197 ps and 1.05 ns, respectively. This may imply that the charge transfer occurring between the Au NPs and ZnO is responsible for the faster decay time components due to exciton–surface plasmon coupling [33–35]. The faster rate of charge transfer from decorated Au NPs to ZnO structures suggested the strong possibility of designing advanced gas sensors and enhancing the photocatalytic activity.

**Figure 4:**
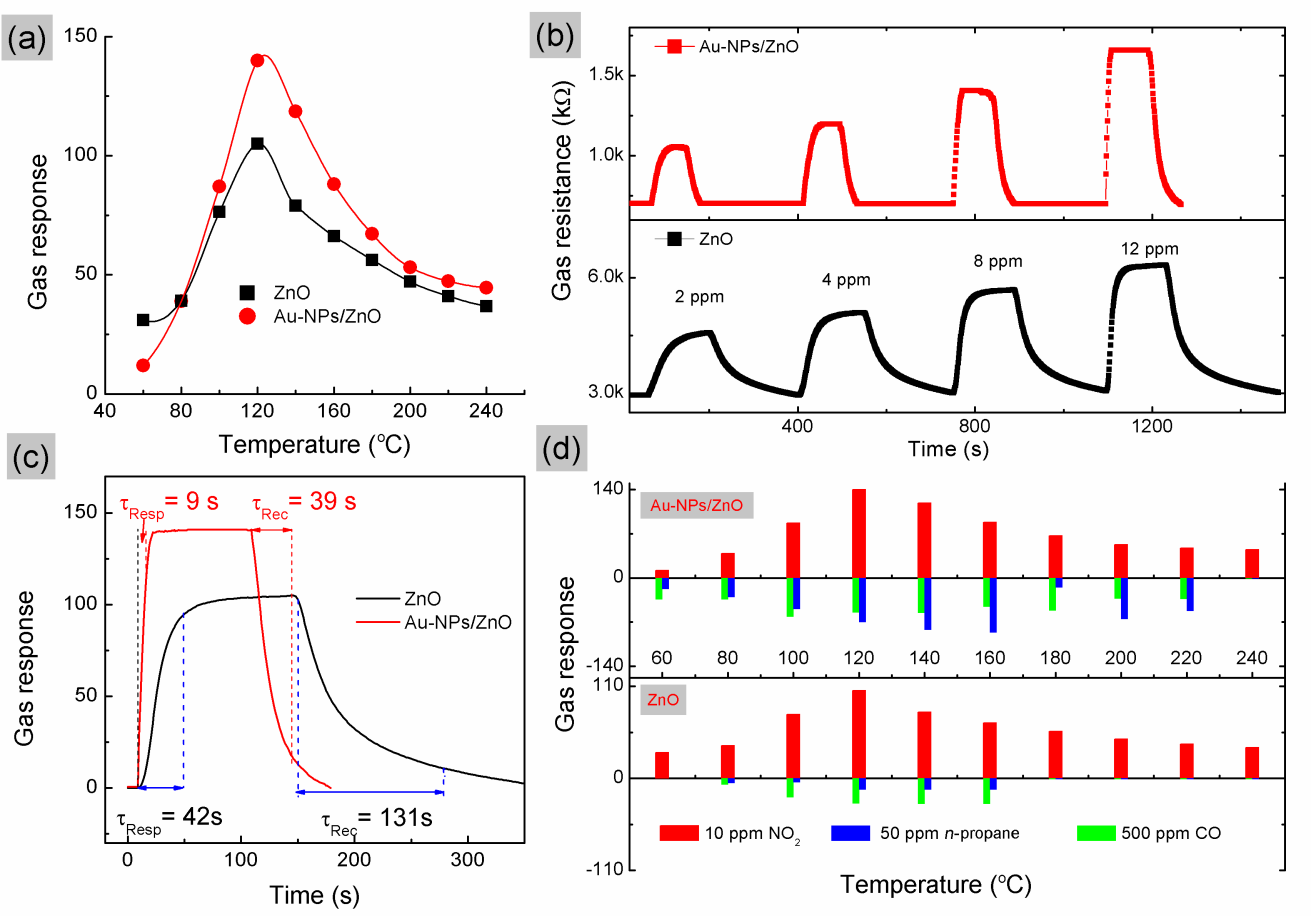
(a) The response of all sensors upon exposure to 10 ppm NO_2_ at different operating temperatures. (b) Dynamic transient of resistance in response to NO_2_ at 120 °C. (c) Response–recovery characteristics and (d) the responses of all sensors for different target gases.

Figure 4a shows the responses of the sensors based on as-deposited samples upon exposure to 10 ppm NO_2_ at different operating temperatures. The optimum operating temperature of both sensors is 120 °C. The maximum sensor response values were found to be 105 and 138 for the ZnO and Au NPs/ZnO sensors, respectively. Figure 4b shows the response and recovery curves of gas sensors upon exposure to 4, 6, 8 and 12 ppm NO_2_ at optimum operating temperature. Interestingly, the sensor response of Au NP/ZnO was fast reaching saturation at concentrations above 8 ppm. The response time (τ_Res_) upon exposure to 10 ppm NO_2_ was dramatically decreased from 42 s to 9 s and the recovery time (τ_Rec_) value was further reduced from 131 to 39 seconds by using Au NP/ZnO structures as expected (Figure 4c).

The response–recovery process, even after five cycles of 10 ppm NO_2_ gas, was observed in Figure S1a (Supporting Information File 1), indicating high reversibility and stability of potential responses in these sensors. In addition, the sensing response of these sensors to the other combustion gases such as CO (500 ppm), and *n*-propane (50 ppm) was also examined and compared to that of NO_2_, as shown in Figure 4d. One can find that both gas sensors are selectively sensitive to NO_2_ gas at the optimum operating temperature of 120 °C. Both ZnO and Au NP/ZnO sensors exhibit too low sensitivity for CO gas, while the presence of decorated Au on the surface of ZnO improves the sensitivity for *n*-propane (C_3_H_8_) detection, which was enhanced from 13.6 to 86.3 times at an operating temperature of 160 °C. The dynamic response transients of both sensors to varying concentrations (15–60 ppm) of *n*-propane at an operating temperature about of 120 °C were also investigated (Figure S1b,c, Supporting Information File 1). These improvements of the sensitivity and the selectivity allow for the detection of a specific gas. We have compared the enhanced performance of our devices to previously reported results of ZnO-based sensing devices toward NO_2_, as shown in Table 1.

**Table 1:** Comparison of sensing performance between our proposed NO_2_ sensors with some previously reported ZnO composites.

Materials	Temp. (°C)	S (*G*/*G*_0_)	NO_2_ gas (ppm)	τ_Res_	τ_Rec_	Ref.

Au/ZnO nanorods	300	10^a^	50	–	–	[36]
5% Eu_2_O_3_-ZnO	300	16^a^	3	3 min	3 min	[37]
Au@ZnO rod-like pristine	300	–	10	336 s	342 s	[38]
ZnO@Au core–shell	150	–	2	108 s	57 s	[39]
ZnO sub-micrometer spheres	120	105^b^	10	42 s	131 s	this work
Au NP/ZnO	120	141^b^	10	9 s	39 s	this work

^a^S = *R*_g_/*R*_a_; ^b^S = [(*R*_g_ – *R*_a_)/*R*_a_ × 100%.

It is known that the conductive gas sensors possess the surface-controlled gas mechanism and the gas response is significantly influenced by the effective surfaces. The adsorbed oxygen species can be easily captured by the electrons from the conduction band and adsorbed onto the surface, leading to a depletion region that broadens again, and thus the electrical resistance of the sensor increases. By decorating the ZnO nanostructures with plasmonic Au NPs, Au–ZnO Schottky junctions were formed [1,14]. Thus, depletion of more carriers from the neighboring surface occurred and the sensor response for the gas detection was enhanced. Furthermore, the enhanced performance of NO_2_ sensing devices can be attributed to the electrons provided by the Au NPs to interact directly with the adsorbed oxygen species and target gases [40–41]. As a result, the gas-sensing properties of Au NP/ZnO sensors are crucial to maintain the high sensitivity and fast response–recovery characteristics.

**Figure 5:**
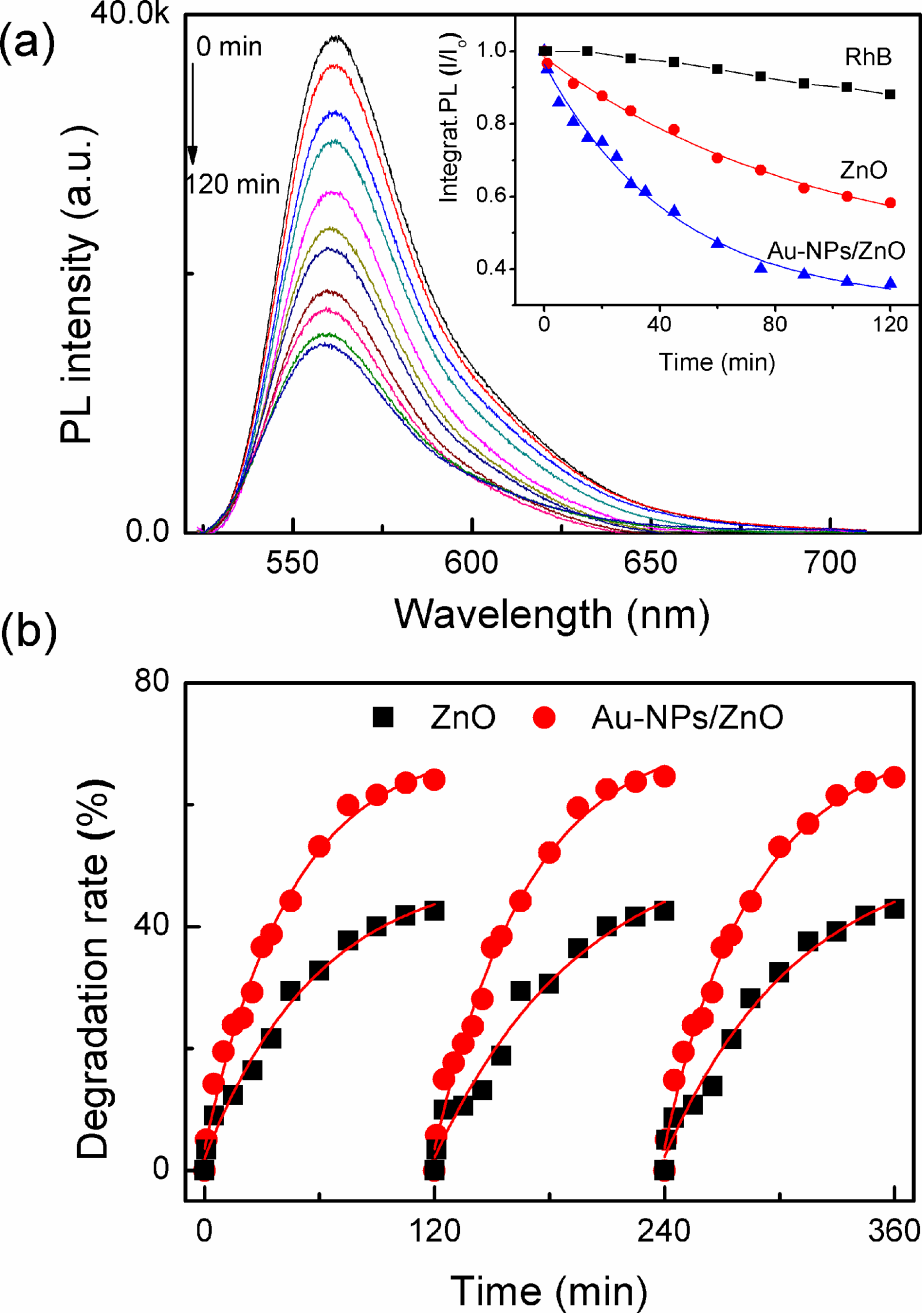
(a) Photoluminescence (PL) spectra of aqueous RhB solution in the presence of as-deposited ZnO and Au NP/ZnO structures under visible-light irradiation. The inset shows the percentage of the PL quenching. (b) Photocatalytic activity of all samples under periodic visible irradiation. The experiment was repeated three times.

The strong electronic interaction between gold and the defect sites of ZnO was assumed to play an important role in improving their photocatalytic performance. In detail, high-energy electrons in Au NPs were injected into the conduction band of the ZnO matrix upon illumination. These electrons then drifted to the conduction band of ZnO, producing active oxygen radicals (e.g., •O^2−^, •OOH, and •OH) at the surface, which are mainly involved in photocatalytic degradation of dyes into CO_2_ or harmless compounds [42]. Therefore, to further determine the ability of Au-decorated ZnO and ZnO sub-micrometer spheres in improving irradiation, PL spectra of an aqueous RhB solution in the presence of these photocatalysts under visible-light irradiation were investigated. The results are shown in Figure 5a and Figures S2 and S3 (Supporting Information File 1).

**Figure 6:**
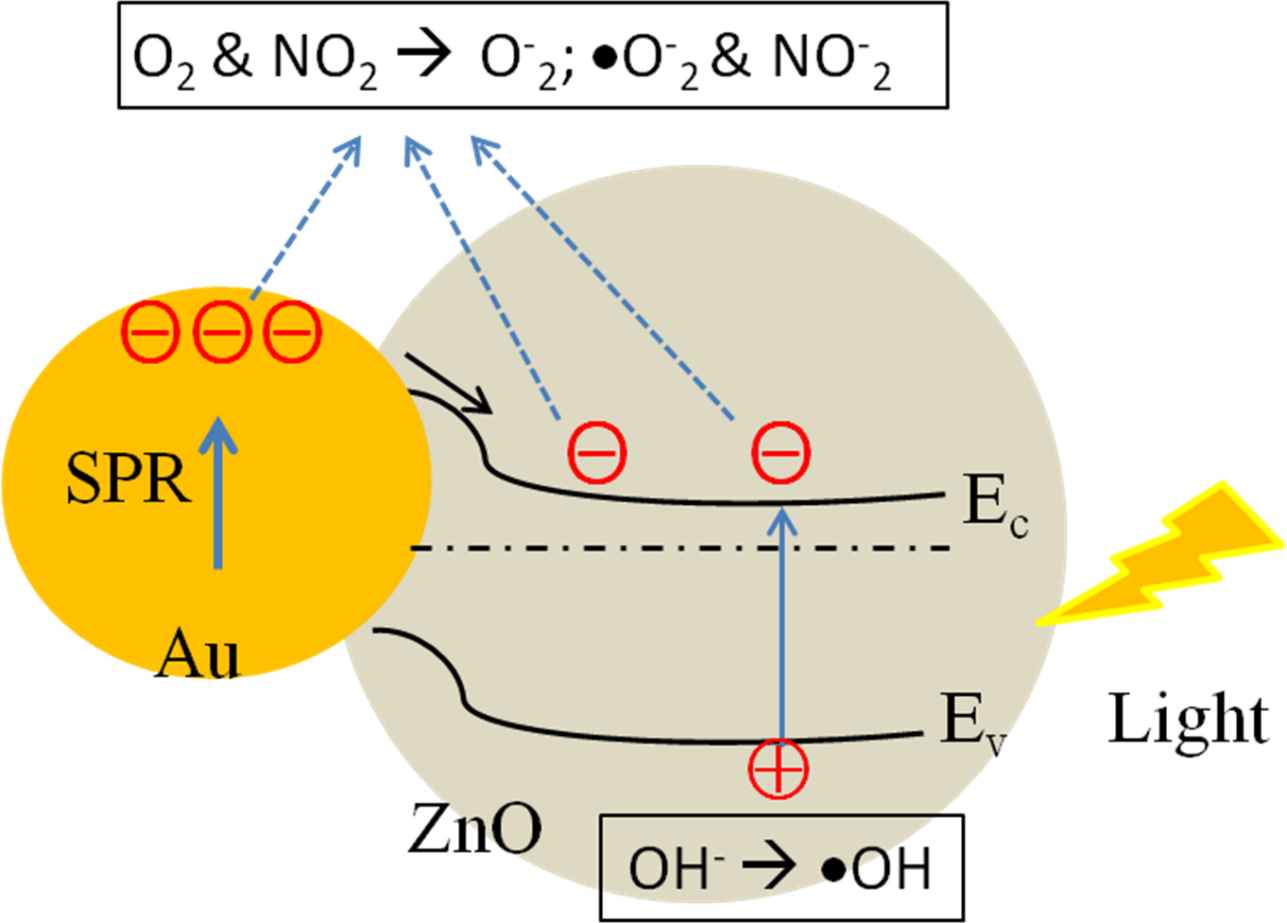
Schematic illustration of the mechanism for the enhanced gas sensing and photoactivity reported for the Au nanoparticle/ZnO structures in this work.

The changes in the PL spectra during photodegradation indicated that the PL peak position of RhB (561.5 nm) decreases with increasing irradiation time. According to the percentage of the PL quenching plotted in the inset of Figure 5a, the degradation efficiency of Au-decorated ZnO is calculated to be 64%, which is higher than ZnO sub-micrometer spheres (42%) after irradiation by visible light for 2 h. Even after three cycles of photocatalytic testing, no significant difference in the photocatalytic degradation process was found, indicating excellent reversibility and stable photocatalytic performance, as shown in Figure 5b. Cascade processes have been previously reported involving the morphology, specific surface and faceted ZnO nano- and microstructures [3,43–44], or trapping of surface and/or defect states [5,42]. In our experiment, the plausible photocatalytic mechanism could be related to the high plasmonic effect of the Au NP/ZnO film in which high-energy photoinduced electrons (hot electrons due to SPR effect with light irradiation) of the Au NPs transfer to the conduction band of the ZnO particle. Thus the Au NPs become as oxidizer for degrading organic pollution substances into CO_2_ or harmless compounds [1,42]. Based on the above interpretation, both the gas sensing and photocatalytic mechanisms can be proposed in terms of adsorption process and reaction of oxygen species and gaseous analytes or degradation molecules on the Au NP/ZnO surface, as illustrated in Figure 6. From these results, we can propose that the plasmonic Au NP/ZnO structures could be a key factor for enhancing the efficiency of photocatalytic decomposition.

## Conclusion

ZnO and hybrid Au NP/ZnO films on glass substrates were synthesized using a simple chemical bath deposition and photoreduction process. The surface plasmonic resonance of the Au NPs on sub-micrometer ZnO spheres was verified by UV–vis absorption, photoluminescence and time-resolved photoluminescence spectra, which in turn supported our study of the design of efficient NO_2_ sensing devices and photocatalytic decomposition of organic dyes. The surface decoration of plasmonic Au nanoparticles has been demonstrated as an effective way to enhance the sensitivity and response–recovery characteristics of NO_2_ gas sensors. The injection electron process from plasmonic Au NPs into the ZnO matrix during light irradiation is the key factor for enhancing the efficiency of the photocatalytic degradation process.

## Experimental

### Synthesis of ZnO and Au-decorated sub-micrometer ZnO spheres

Glass microscope slides were cleaned in ethanol and deionized water in an ultrasonic bath for 3 min, and then dried at 80 °C. In order to obtain the ZnO films, typically, a portion of 10 mL of a 0.1 M aqueous solution of zinc acetate dihydrate [Zn(CH_3_COO)_2_·2H_2_O], and 20 mL of mono-ethanolamine (MEA) were added into a flask containing water (100 mL). The pH was adjusted with ammonia solution. The substrate had to be placed upright in solution. Synthesis was performed at 90 °C for 3 h, with magnetic stirring, and the final pH was typically 10.8–11. After cooling to room temperature, the sub-micrometer ZnO spheres on the glass substrates were washed several times with deionized water, ethanol and dried at 80 °C.

For incorporation of gold nanoparticles, a 0.025 mM aqueous solution of HAuCl_4_ was used. The ZnO films were immersed into this precursor solution. The reaction mixture was constantly irradiated by using a halogen lamp (150 W, Osram) at room temperature. After reacting for 10 h, the Au-decorated ZnO film was washed with deionized water and ethanol and then dried at 80 °C for 12 h. Finally, both prepared samples (ZnO and Au NP/ZnO) were heat-treated at 400 °C for 30 min in air to remove all remaining solvent and residual impurities before carrying out further characterization.

### Structural characterization

The crystalline structure and morphology of the as-deposited sub-micrometer ZnO spheres were characterized by X-ray diffraction (XRD, Bruker D8 Advance), SEM (FE-SEM, HITACHI S-4800), and HRTEM (JEOL2100). UV–vis analysis was carried out on a spectrophotometer (FLAME-S, Ocean Optics, Inc.). PL measurements (IK3301R-G, Kimmon Koha) were performed at room temperature using a He–Cd laser source (325 nm). For TRPL decay measurement of ZnO structures, a time-correlated single photon counting (TCSPC) technique was used. We used 350 nm frequency doubled femtosecond pulses from a 76 MHz mode locked Ti:sapphire laser system as a light source. The PL was collected by using lenses, and then dispersed by a 15 cm monochromater, and finally detected by a UV enhanced multichannel plate photomultiplier tube (MCP-PMT). The full-width at half-maximum (FWHM) of the total instrument response function (IRF) was less than 130 ps.

### Gas sensing characteristics

Gas sensing properties of the as-deposited ZnO and Au NP/ZnO samples were evaluated by measuring the resistance change of the corresponding sensors using two Pt electrodes, which were deposited on the surface of the ZnO and Au NP/ZnO films. The gas sources included NO_2_, CO, and C_3_H_8_ (Singapore Oxygen Air Liquide Pte., Ltd) and were used for analyzing the gas sensing characteristics. The sensors were investigated in a chamber of 50 mL in volume, and the total gas flow rate through the chamber was fixed at 500 mL/min. The sensor response is defined as [*R*_g_ – *R*_a_]/*R*_a_ × 100%, where *R*_g_ and *R*_a_ are the resistances of the ZnO-based sensors tested in NO_2_ gas and in clean air, respectively.

### Photocatalytic degradation test

The photocatalytic activity wase evaluated by the photodegradation of an aqueous rhodamine B (RhB) solution at room temperature. Experiments were performed as follows: the prepared ZnO and Au NP/ZnO films (7.5 × 7.5 mm) were added into 2 mL of an aqueous RhB solution (1 × 10^−6^ M) in a quartz cuvette, and then the cell was ultrasonicated for 5 min to make the homogeneous distribution of photocatalyst with RhB. Before light irradiation, the cell containing the photocatalyst and the aqueous RhB solution was kept in the dark for 40 min to reach an adsorption/desorption equilibrium between the photocatalyst and RhB molecules. The RhB solution was illuminated with a halogen lamp (150 W) at a distance of 60 cm. After irradiating for a period (every 15 min), the PL spectra of the RhB solutions after photocatalytic activation were measured by a spectrophotometer (FLAME-S, Ocean Optics, Inc.) using the excitation source of a 405 nm laser. The photocatalytic decomposition of RhB molecules is evaluated by the PL quenching of the RhB in the photocatalyst-mixed solution. The percentage of the PL quenching is calculated by the ratio of integrating the measured PL emission from 500–700 nm.

## Supporting Information

File 1 Additional spectra.
