# Supplementary material for: Surface-plasmon-enhanced ultraviolet emission of Au-decorated ZnO structures for gas sensing and photocatalytic devices
Source: Beilstein J Nanotechnol. 2018 Mar 1;9:771–9. doi: 10.3762/bjnano.9.70 (PMC5852533; doi:10.3762/bjnano.9.70)
Supplement: File 1 — Additional spectra. [file Beilstein_J_Nanotechnol-09-771-s001.pdf]

Supporting Information

for

Surface-plasmon-enhanced ultraviolet emission of Au-decorated ZnO structures for gas sensing and photocatalytic devices

T. Anh Thu Do[1*§], Truong Giang Ho[1], Thu Hoai Bui[2], Quang Ngan Pham[1], Hong Thai Giang[1], Thi Thu Do[1], Duc Van Nguyen[1] and Dai Lam Tran[3]

Address: [1]Institute of Materials Science, Vietnam Academy of Science and Technology, 18 Hoang Quoc Viet, Caugiay, 100000, Hanoi, Vietnam, [2]Petro Vietnam University, 762 Cach Mang Thang 8, Longtoan, 790000, Ba Ria-Vung Tau, Vietnam and [3]Graduate University of Science and Technology, Vietnam Academy of Science and Technology, 18 Hoang Quoc Viet, Caugiay, 100000, Hanoi, Vietnam

*Corresponding author

Email: T. Anh Thu Do - thudta@ims.vast.vn

[§]Tel.: +84-243-7569318; Fax: +84-243-8360705

Additional spectra

I. Gas sensing characteristics

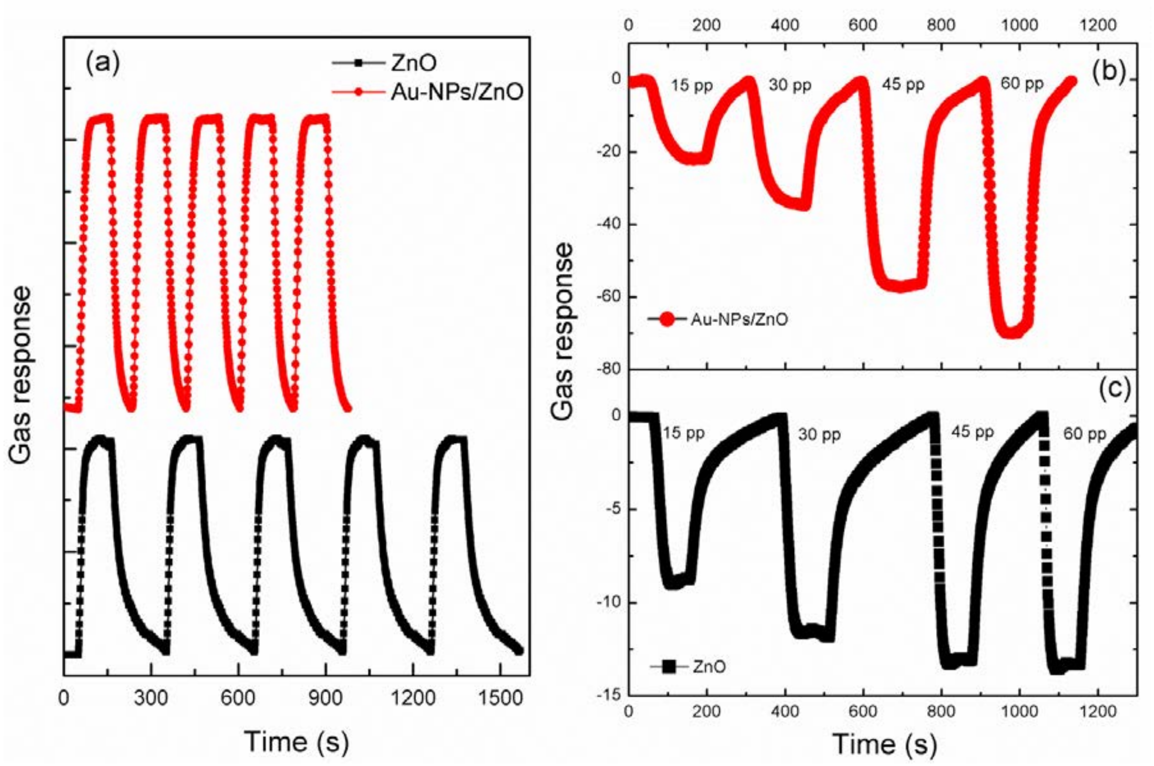

Figure S1: (a) Response cycles of all samples to 10 ppm NO_2 at operating temperature (120 °C); (b) and (c) dynamic transient of resistances in response to *n*-propane (C_3H_8) detection for ZnO and Au-NPs/ZnO sensors at 120 °C, respectively.

II. Photocatalytic degradations

The decoloration percentage which demonstrates the photocatalytic activity of both samples is evaluated by the PL quenching of RhB in the photocatalyst-mixed solution, which is determined by the expression,

$$\text{PL quenching } (\%) = \frac{(\mathrm{I}_0 - \mathrm{I(t)})}{\mathrm{I}_0} \times 100\%$$

where I_o and I(t) are the integrated PL intensity of RhB solution before and after visible light irradiation, respectively.

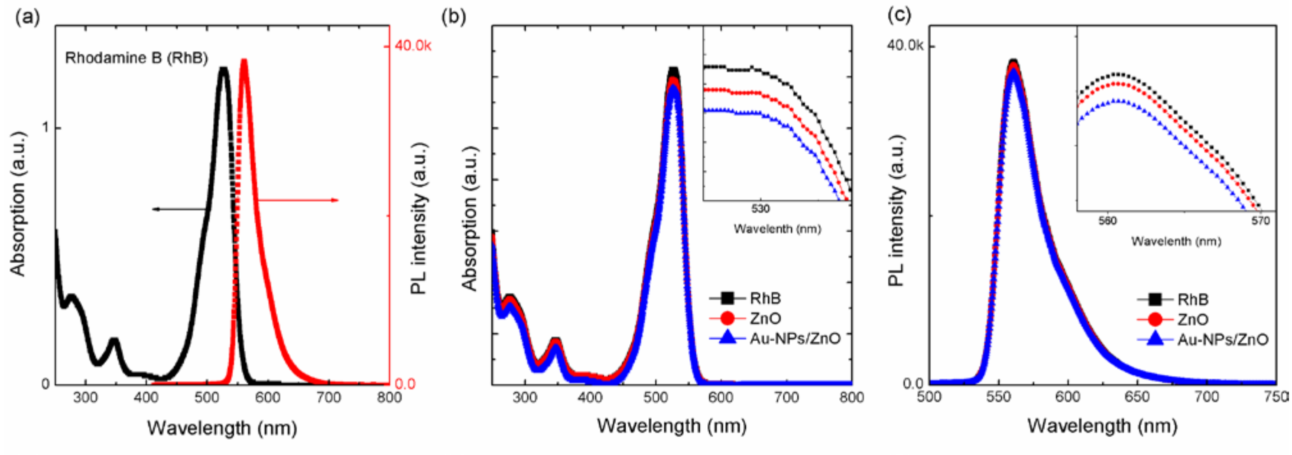

Figure S2: (a) UV–vis absorption and PL spectrum of Rhodamine B (RhB) aqueous solution at room temperature; (b) UV–vis absorption spectra; and (c) PL spectra of all samples, indicating slight decreases in the photo-decomposes even after 40 min (the inset of b and c).

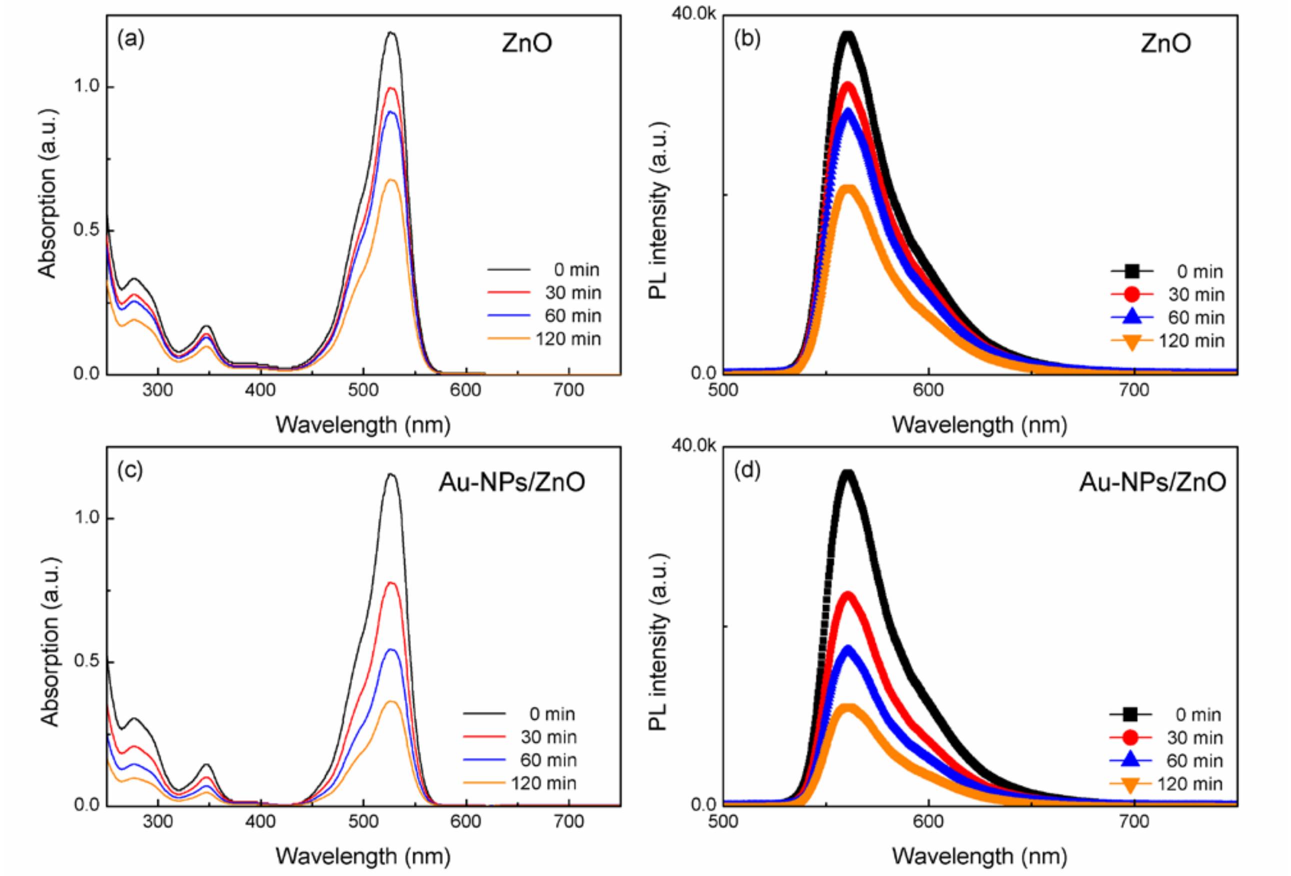

Figure S3: UV–vis absorption and PL spectra of RhB aqueous solution in presence of as-deposited ZnO film (a, b), and ZnO/Au film (c, d), respectively, after different periods of time.
